# Genomic diversity and population structure of Carniolan honey bee in its native habitat

**DOI:** 10.1186/s12864-024-10750-z

**Published:** 2024-09-10

**Authors:** Boris Lukic, Nikola Raguz, Marin Kovačić, Ino Curik, Jana Obšteter, Janez Prešern, Jernej Bubnič, Ras Lužaić, Ivan Pihler, Goran Mirjanić, Marco Pietropaoli, Zlatko Puškadija

**Affiliations:** 1 Faculty of Agrobiotechnical Sciences Osijek, J.J. Strossmayer University of Osijek, Vladimira Preloga 1, Osijek, 31000 Croatia; 2 Department of Animal Science, Faculty of Agriculture, University of Zagreb, Svetošimunska cesta 25, Zagreb, 10000 Croatia; 3 Department of Animal Science, The Agricultural Institute of Slovenia, Hacquetova ulica 17, Ljubljana, 1000 Slovenia; 4 Department of Animal Science, Faculty of Agriculture, University of Novi Sad, Trg Dositeja Obradovića 8, Novi Sad, 21000 Serbia; 5 Faculty of Agriculture, University of Banja Luka, 1A Vojvode Petra Bojovića Blvd, Banja Luka, 78 000 Bosnia and Herzegovina; 6 L’Istituto Zooprofilattico Sperimentale del Lazio e Toscana, Via Appia Nuova 1411, Roma (Capannelle), 00178 Italy; 7 Institute of Animal Sciences, Hungarian University of Agriculture and Life Sciences, 40, Guba S. str, Kaposvár, H-7400 Hungary

**Keywords:** *Apis mellifera carnica*, Genomic diversity, Population structure, Single nucleotide polymorphism (SNP), Beekeeping, Conservation

## Abstract

**Background:**

Research into the genetic diversity of honey bee (*Apis mellifera* *L.*) populations has become increasingly significant in recent decades, primarily due to population declines attributed to human activities and climate change. As a species of great importance, breeding programs that leverage understanding of genomic diversity could offer solutions to mitigate these challenges. The objective of this study was to examine the genomic diversity and population structure of Carniolan honey bees (*Apis mellifera carnica*) using the Illumina SNP chip on a large honey bee sample collected from Central and South-Eastern European countries. The study also aims to offer recommendations for future breeding programs.

**Results:**

Our analysis involved Discriminant Analysis of Principal Components (DAPC), heterozygosity, admixture analysis, fixation indices (F_ST_), Neighbour-Joining tree, gene flow and Isolation-by-distance analysis. DAPC indicated distinct separation between the Carniolan and Italian honey bee (*Apis mellifera ligustica*) populations, whereas the admixture analysis revealed varying levels of gene flow and genetic admixture within the Carniolan honey bee populations, demonstrating closer relationships between specific geographic regions (confirmed by Isolation-by-distance analysis). Furthermore, the research of heterozygosity, genomic inbreeding, pairwise F_ST_ values, and Neighbour-Joining tree provided insights into the patterns of genetic differentiation and similarity among the populations of Carniolan honey bee within its natural habitat. We have observed genetic homogeneity of the Carniolan honey bee population when considered in a broader genetic/geographical context. However, the Carniolan honey bee has sufficient genetic diversity in its geographical home range that needs to be carefully monitored and maintained.

**Conclusions:**

This study provides important insights into the genetic composition, differentiation, and relationships among Carniolan honey bee populations in Central and South-Eastern European countries. The findings are crucial for conservation efforts, breeding programs, and sustainable beekeeping practices. They emphasise the importance of considering genetic factors and population structure in the breeding and management of honey bees. By understanding these genetic relationships, we can develop strategies to preserve genetic diversity, improve breeding outcomes, and ensure the resilience of honey bee populations in the face of environmental changes and challenges. This knowledge can also inform policy makers and stakeholders on best practices to maintain healthy bee populations, which are vital for ecosystem services and agricultural productivity.

**Supplementary Information:**

The online version contains supplementary material available at 10.1186/s12864-024-10750-z.

## Background

In the last few decades in Europe, research on genetic diversity of honey bee (*Apis mellifera* L.) populations became increasingly important, mainly due to the population decline caused by excessive use of pesticides, spread of mite *Varroa destructor,* along with climate changes [1–4] and human activities [5]. Although the colony number is growing (which is result of more people becoming honey producers), in some European countries (UK, Scandinavian and Baltic), population decline was estimated to more than 30% in 2012–14 [6, 7], whereas in 2017–18 average decline in Europe was 16.4% [8], therefore the reduction of genetic diversity is expected to become higher. In addition, more productive subspecies such as *Apis mellifera carnica* (AMC) in certain European countries are being preferred by the breeders [5], which could lead to more homogeneous and less diverse genetics. Likewise, a consequence of uncontrolled human activities such as introgressive hybridisation, could negatively influence genetic diversity [9]. Because of these circumstances, it is crucial to analyse genetic diversity of local honey bee populations and provide recommendations for future breeding and conservation purposes.

In Central and South-Eastern Europe, the AMC honey bee is an indigenous subspecies found in the region bordered by the Carpathian Mountains to the north, the Alpine mountains to the west, the Adriatic coast, and the Prokletije mountains to the southeast [10]. Classified under the evolutionary lineage C, this subspecies was characterized in the seventies using a multifactorial analysis of 33 morphometric phenotypes [10]. Since then, several studies based on the phenotypic data characterized and confirmed [11–13] the initial Ruttner’s characterization of Carniolan honey bee in relation to the other *A. mellifera* subspecies. The latest study [14] of wings geometric morphometry on samples of honey bees from Croatia and Slovenia showed that majority of their wing phenotypes are similar to the AMC from the historical datasets of former Yugoslavia and Austria, and somewhat different from the populations from Hungary, Romania, and Greece.

In addition to phenotypic characterization, numerous studies based on the genomic data were performed in order to achieve more accurate subspecies characterization and gain deeper insights into genetic diversity. The population diversity of Carniolan honey bee was analysed using mitochondrial DNA (mtDNA) with RFLPs as well as DNA microsatellites [15]. One mtDNA haplotype (COI-COII region) was detected in the samples of Slovenian and Croatian honey bees, indicating their genetic affiliation to the same population, albeit with a limited Croatian sample size (*N* = 10). Also, based on their results, they indicated possible level of crossbreeding with AMC population from the Czech Republic. Another study on mtDNA (COI-COII region) and microsatellites on Croatian (*N* = 20), Italian, and Greek populations [16], detected two subpopulations in Croatia and certain level of admixture with Greek and Italian populations. Recent study on AMC population from Serbia using microsatellites of nuclear DNA [17], suggests relatively homogeneous genetic structure and clear distinction from the neighbouring *A. m. macedonica* subspecies. Based on the mtDNA analyses [18, 19], two additional haplotypes were detected in Serbian population, therefore higher genetic diversity was indicated. Nevertheless, these reports hint at high level of genetic variation present in the Central and South-Eastern European Carniolan honey bee populations, which should be carefully conserved in national breeding programs. However, the majority of the previously described studies on Carniolan honey bees were based on the mtDNA and microsatellite markers, which were shown to be imprecise in the context of detection of population diversity, incompatible for comparison between studies, and usually require high sample size.

In the last ten years, with the advent of SNP microarrays based on high-throughput genotyping technologies, thousands of genotypes became commercially available for the majority of livestock species, therefore their application in genetic diversity analyses and genomic selection became state of the art technology. Recently developed SNP panel for honey bees [20] allows identification of population genomic parameters such as genomic relationship, genetic admixture, F_ST_ etc. Although some studies recommend only 50 [21], 95 [22] or 153 [23] SNPs for genomic conservation of honey bees, the aforementioned SNP chip was used. It was specifically designed by using machine learning algorithms which selected over 4000 highly informative markers to assure accurate classification of new honeybee subspecies. Activities aimed at preventing the loss of genetic diversity will be way more optimized if the parameters of genomic population structure are properly analysed and finally well understood. Therefore, monitoring parameters becomes crucial, particularly in the era of climate changes and new challenges in species adaptation [24]. It is essential to preserve all the variability in the population to sustainably breed bees that can adapt to a changing environment. The aim of this study was to (i) analyse population structure by computing genomic relationship, genetic admixture, gene flow, genomic inbreeding, fixation index (F_ST_), Nei’s genetic distances and Isolation-by-distance using honey bee SNP BeadChip on large Central and South-Eastern European Carniolan honey bee sample, covering three Croatian regions, Slovenia, Hungary, Serbia, Bosnia and Herzegovina, Montenegro, and Italy, and (ii) to provide recommendations for AMC breeding programs.

## Methods

### Populations and sampling

**Fig. 1:**
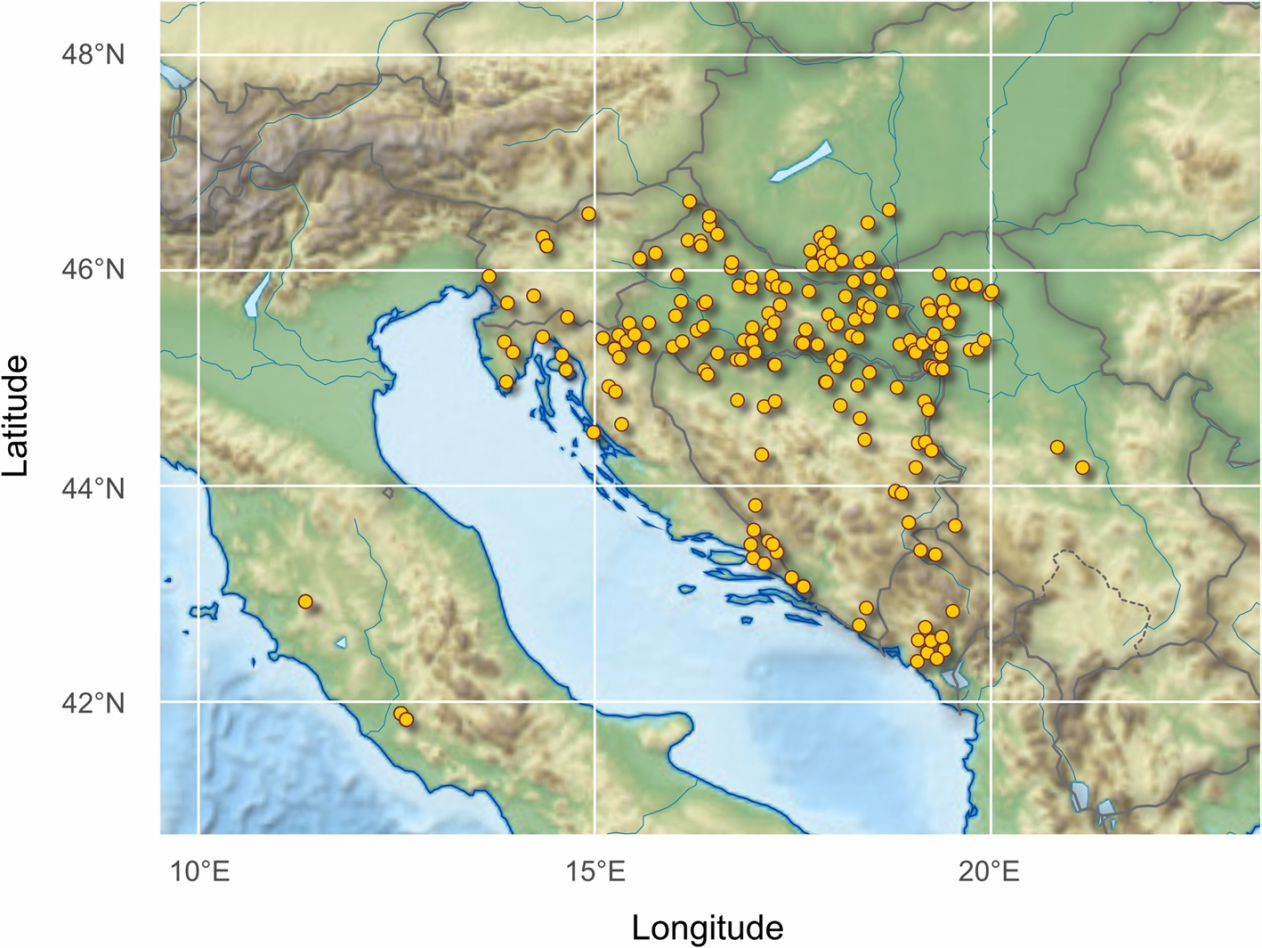
Geographical distribution of the honeybees analysed in this study

In order to obtain sufficient representation of AMC honey bee populations in the Central and South-Eastern Europe, we obtained large collection of 232 worker bee samples (each from a single colony per one stationary apiary), distributed across six neighbouring countries representing a continuous territory: Croatia – CRO (represented with three regions: Continental - CROC, *n* = 35; Subalpine - CROS, *n* = 42; and Adriatic - CROA, *n* = 26), Slovenia - SLO (*n* = 18), Serbia - SRB (*n* = 29), Bosnia and Herzegovina - BIH (*n* = 29), Montenegro - MNE (*n* = 11), Hungary - HUN (*n* = 17) and samples of *Apis mellifera ligustica* (AML) from Italy - ITA (*n* = 25) as an outgroup (Fig. 1). Overall, eight populations were analysed as AMC. In this study, the dataset from Croatia and Slovenia underwent prior morphometric analysis [14]. To ensure a balanced sample size per country or region, it was reduced from 160 samples to 103, thus avoiding overrepresentation. Additionally, the dataset was expanded to include populations from other countries as previously mentioned for the purposes of this research.

**Fig. 2:**
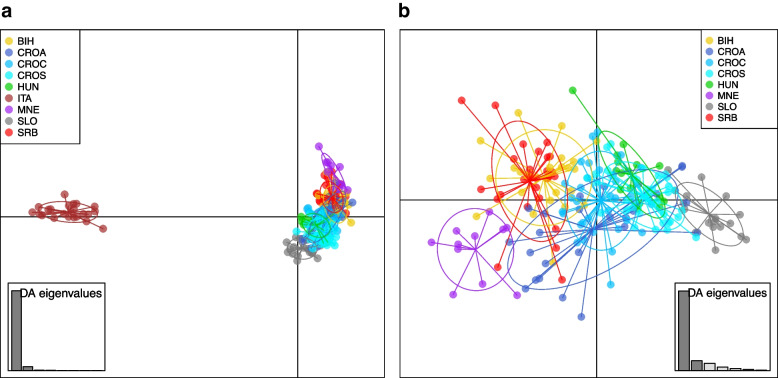
Graphical illustration of the population structure presented by the first two discriminant functions generated by “supervised” Discriminant Analysis of Principal Components (DAPC) algorithm **a**) Scatter-plot related to *Apis mellifera carnica* and an outlying *Apis mellifera ligustica* population; **b** Scatter-plot related only to *Apis mellifera carnica* populations

The samples were collected on apiaries owned by beekeepers that do not buy queens or swarms on the market. In this way, we were able to provide realistic results about the genetic structure of local populations. More detailed information about the samples and populations is available in the Additional file 1. After collection in the field, samples of the whole honey bees were immediately preserved in 96% ethanol and subsequently stored in the freezer at -20 °C. Genomic DNA was extracted from thorax using isolation kit and genotyped using 4 K Illumina Infinium BeadChip with 4165 SNPs [20]. SNP density of the used SNPchip is available in the Additional file 2. Quality control of the genomic data was performed in PLINK [25]. SNPs where more than 5% of genotypes were missing and SNPs with Illumina GenCall score ≤ 0.5 were excluded from the analysis. Worker bees for which > 10% of the genotype was missing were also excluded from further analysis. SNP positions were based on the honey bee reference genome assembly Amel 4.5. After quality control, 3283 SNPs and 212 samples were left for the analysis. Discriminant Analysis of Principal Components (DAPC) was performed in R [26] package adegenet [27]. At the beginning of the analysis, we identified almost 200 PCs explaining our dataset. To determine the optimal number of clusters, we ran *k*-means approach sequentially with four different scenarios with 10, 30, 50, and 100 retained components, respectively, and the final clustering solutions (Additional file 3) were compared using the Bayesian Information Criterion (BIC). We assumed a maximum of 10 clusters, which is slightly higher than the actual number of eight populations analysed. As expected, the number of retained components has considerable impact on the number of clusters identified, and based on this analysis, 3–4 possible clusters were identified in our dataset. In addition to *k*-means, we used discriminant analysis (DAPC function) with the same scenarios of 10, 30, 50 and 100 retained components (solutions are available in Additional file 4). To prevent overfitting, we calculated the a-score, which measured the difference between the proportion of observed discrimination and the values obtained through random discrimination. The results, detailed in Additional File 5, covered scenarios with 10, 30, 50, and 100 retained components. It was evident that the optimal number of retained components was around 15, which we used for the final analysis (Fig. 2).

### Population structure and differentiation of populations

To provide estimates of genetic diversity within honey bee populations we calculated expected/observed heterozygosity and the population inbreeding (F_POP_) coefficients based on their ratio using PLINK [25]. This F_POP_ is equivalent to F_IS_ of Wright’s F-statistics. Genetic differentiation between six investigated honey bee populations and one outgroup AML population from Italy was performed by the genome wide fixation index, F_ST_, for each SNP pair [28] calculated in PLINK [25]. In addition, we analysed genetic divergence among populations based on Nei’s distances matrix [29] and visualized it by the Neighbour-Joining tree (NJ) using SplitsTree4 software [30]. Nei genetic distances were calculated in R using package stAMPP [31].

### Unsupervised analysis of population structure and genetic admixture

The unsupervised analysis of population structure and genetic admixture was conducted on the final data set (AMC and AML) using a Bayesian clustering approach implemented in STRUCTURE v.2.3.4 [32] to enhance the reliability of our results. We employed an admixture and correlated allele frequency model, testing from one to eight ancestral populations. For each K value, eight runs of 100,000 Markov chain Monte Carlo iterations were performed following a burn-in period of 10,000 iterations. The visualization of the results and determination of the most likely number of clusters were carried out using the ΔK method in Structure Selector software [33, 34].

### Gene flow

Contemporary gene flow between honey bee populations was analysed by assessing migration rates (m) using the BayesAss v3.0 assignment test [35]. The evaluation consisted of 20 replicates with different random seeds, each subjected to Markov chain Monte Carlo simulations with up to 22 million iterations, discarding the first two million iterations during the burn-in process. The mixing parameters—delta allele frequency, delta migration rate, and delta inbreeding coefficient—were set to 0.1. Ten of the replications included the AML population, while the remaining ten were performed without AML. The resulting “log outputs” were analysed using Tracer [36], and Bayesian deviance was computed in R. Migration estimates from the three runs with the lowest Bayesian deviance were merged to create a posterior distribution encompassing the estimated migrations.

### Isolation by distance

In order to evaluate the effect of Isolation by distance of AMC, Mantel test [37] was performed in R package ade4 [38] by regressing pairwise genetic distances [29] against the Euclidean geographical distances. The significance of the empirical correlation between the genetic distance matrix and geographic distance matrix was assessed through 1000 Monte Carlo simulations in the scenario of absence of spatial structure.

## Results

### Discriminant analysis of principal components (DAPC)

In order to analyse genetic relationship of the AMC, DAPC approach was applied to calculate genetic population structure between all collected honey bee populations, as shown in Fig. 2a and 2b. As expected, the first and second discriminant functions clearly separated the two subspecies AMC and AML into two main clusters (the calculated discriminant functions accounted for 31.4% of the total conserved variance). In the same analysis, the second discriminant function separated the AMC populations (Fig. 2a).

The results of a deeper insight into the DAPC, performed only for the AMC populations, show that the central part of the AMC consists of the populations of CROA, CROC, CROS, HUN, BIH and SRB (the latter two tend to cluster together), while the populations of MNE and SLO, which are geographically on the border, slightly overlap with the main cluster of the AMC (Fig. 2b). The most differentiating SNPs with highest loading values are available in Additional file 6. However, this analysis (the calculated discriminant functions accounted for 23.7% of the total variance obtained) also showed a certain dispersion of samples from all AMC populations with the exception of the Montenegrin and Slovenian populations. As the Croatian territory is generally very diverse and includes different specific environments (continental, mountainous and Adriatic) that are important for honey bee adaptation, DAPC was performed separately for three Croatian populations (labelled CROA, CROC and CROS, Additional file 7). The results showed that the two historically considered subpopulations, the continental and subalpine populations, are actually a single population, while CROA bees were scattered across the AMC cluster, albeit to a very small extent that is not sufficient to characterise them as a separate subpopulation (Fig. 2b). However, all Croatian populations were found to have some degree of dispersal, depending on where they were sampled.

### Population diversity and relationships

The values of observed heterozygosity (H_O_) and expected heterozygosity (H_E_) are given together with the F_POP_ values, which indicate the level of genomic inbreeding of the populations. The analysis of heterozygosity revealed varying levels of genetic diversity among the honey bee populations. MNE displayed the highest genetic diversity, while the Italian population showed the lowest. Low to moderate genetic diversity was observed in other populations, while relatively similar levels were found in CRO, HUN, SLO and SRB. The population inbreeding coefficients can be interpreted based on the observed and expected heterozygosity values provided in Table 1. The inbreeding coefficient (F_POP_) represents the deviation of the observed heterozygosity from the expected heterozygosity in Hardy–Weinberg equilibrium. In populations with negative F_POP_ values (e.g. SRB, HUN, BIH), the observed heterozygosity exceeds the expected values. This indicates a potential increase in genetic diversity within these populations, probably due to the gene flow observed in this study or to outbreeding as an alternative possibility. In contrast, highest F value was detected in CROA. However, it is crucial to note the presence of relatively large standard errors in our analysis of F_POP_. These standard errors indicate the confidence of our estimates, revealing that the estimated negative or positive F_POP_ values are not significantly different from zero due to the wide confidence intervals.

**Table 1:** Genetic diversity among honey bee populations based on heterozygosity

Population	H_O_ ± SE	H_E_ ± SE	F_POP_
Serbia - SRB	0.2559 ± 0.004	0.2508 ± 0.004	-0.012 ± 0.020
Hungary - HUN	0.2466 ± 0.004	0.2403 ± 0.004	-0.020 ± 0.027
Bosnia & Herzegovina - BIH	0.2621 ± 0.004	0.2577 ± 0.004	-0.013 ± 0.020
Montenegro - MNE	0.3410 ± 0.005	0.3171 ± 0.005	-0.059 ± 0.028
Italy - ITA	0.1933 ± 0.003	0.1923 ± 0.003	-0.003 ± 0.037
Slovenia - SLO	0.2466 ± 0.004	0.2407 ± 0.004	-0.020 ± 0.018
Croatia Adriatic - CROA	0.2432 ± 0.004	0.2500 ± 0.003	0.033 ± 0.021
Croatia Continental - CROC	0.2314 ± 0.004	0.2330 ± 0.004	0.010 ± 0.016
Croatia Subalpine - CROS	0.2207 ± 0.004	0.2208 ± 0.004	0.003 ± 0.012

Genetic diversity indices for AMC and AML. H_O_, observed heterozygosity; H_E_, expected heterozygosity; F_POP_, population inbreeding coefficient

The population differentiation across all honey bee populations was analysed by pairwise F_ST_ values, as shown in Table 2. The mean F_ST_ estimate was 0.088 including the AML, while only between the AMC populations, mean F_ST_ value was 0.061. The F_ST_ values ranged from 0.000 (between the SRB and BIH) to 0.359 (between the MNE population and AML). Comparison among the AMC populations only, showed highest differentiation between MNE and HUN (0.068) and SLO and SRB (0.049). Genetic differentiation between populations tends to be lower when they share a closer genetic history. In this study, the low F_ST_ values observed among the AMC populations indicate a high degree of genetic similarity, suggesting recent common ancestry or continuous gene flow. This low level of genetic differentiation highlights the potential for these populations to share similar genetic traits and adaptive characteristics. Understanding genetic relatedness within AMC is crucial for conservation and breeding strategies, as it can guide efforts to maintain genetic diversity and resilience. Moreover, this knowledge can inform management decisions to ensure the genetic integrity of these populations in the face of environmental change and human encroachment.

**Table 2:** Genetic differentiation between honey bee populations based on F_ST_ estimates

Population	SRB	HUN	BIH	MNE	ITA	SLO	CROA	CROC	F_ST_
Serbia - SRB									**0.055**
Hungary - HUN	0.017								**0.053**
Bosnia & Herzegovina - BIH	0.000	0.015							**0.055**
Montenegro - MNE	0.024	0.068	0.029						**0.094**
Italy - ITA	0.314	0.283	0.322	0.359					**0.305**
Slovenia - SLO	0.049	0.014	0.046	0.117	0.275				**0.072**
Croatia Adriatic - CROA	0.009	0.022	0.005	0.024	0.309	0.043			**0.055**
Croatia Continental - CROC	0.008	0.003	0.005	0.051	0.292	0.019	0.008		**0.049**
Croatia Subalpine - CROS	0.021	0.004	0.017	0.078	0.286	0.012	0.019	0.003	**0.055**

Pairwise F_ST_ estimates (Weir and Cockerham 1984) presenting genetic differentiation among nine selected populations and average F_ST_ estimates for each population (right column)

**Fig. 3:**
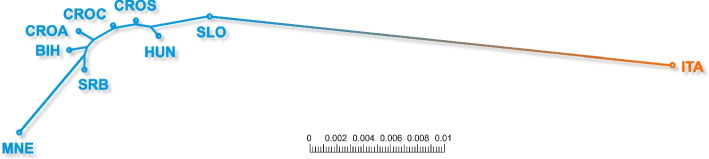
Neighbour-Joining tree based on Nei’s genetic distances (total scale = 0.01) for the eight *Apis mellifera carnica* (blue) populations (CROA - Adriatic Croatia; CROC - continental Croatia; CROS - subalpine Croatia; BIH - Bosnia and Herzegovina; HUN - Hungary; MNE - Montenegro; SLO - Slovenia and SRB - Serbia) and an outlying population of *Apis mellifera ligustica* (orange)

The genetic structure of AMC populations is illustrated by a Neighbour-Joining tree constructed from pairwise Nei’s genetic distances, as shown in Fig. 3. Nei’s standard genetic distance, one of the most commonly used genetic distance measures, assumes mutation and drift as the main forces shaping population divergence [29]. This measure is particularly suitable for graphical illustration of population structure using a Neighbour-Joining tree.

As can be seen from Fig. 3, the most distant AML population from Italy (outlier) is connected to the eight analysed Carnica populations through the Slovenian population, while the Carnica population furthest away from the Italian AML population was the one from Montenegro. This result is consistent with the F_ST_ values in Table 2. On the other hand, the other six AMC populations were grouped in a consecutive order with small distances: HUN, CROS, CROC, CROA, BIH, and SRB. This strongly suggests that the Adriatic Sea represents a significant geographical barrier to gene flow between the neighbouring AML and AMC subspecies, which diverged a long time ago [39]. Despite the small differences between the distances, we were surprised that HUN was closer to ITA and SLO than to any of the Croatian populations (CROS, CROC and CROA).

### Unsupervised population structure and admixture analysis

**Fig. 4:**
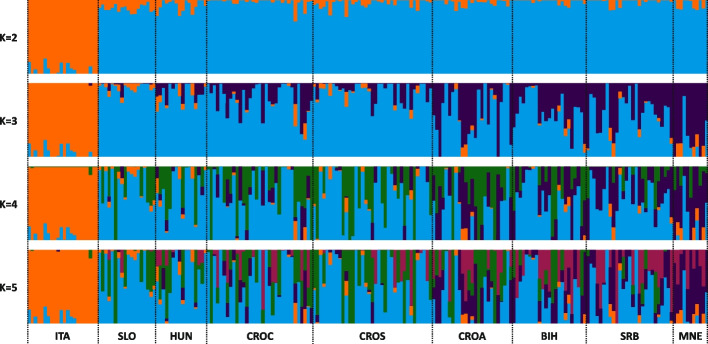
Graphical representation of the results of the unsupervised population structure and admixture analysis using the STRUCTURE algorithm for 212 bees from one AMC population (ITA - Italy) and eight AML populations (CROA - Adriatic Croatia; CROC - continental Croatia; CROS - subalpine Croatia; BIH - Bosnia and Herzegovina; HUN - Hungary; MNE - Montenegro; SLO - Slovenia and SRB - Serbia), selection of results for K = 2, K = 3, K = 4 and K = 5. Each individual is represented by a vertical line coloured on the basis of admixture proportions, reflecting ancestral genetic contributions

The genetic structure of eight AMC and one AML population determined using the STRUCTURE algorithm is shown in Fig. 4.

The results of the STRUCTURE analysis in relation to the first split with K = 2 were as expected and show that there is a very low level of admixture between AMC and AML, with this low level of admixture remaining constant regardless of the higher K-s. By observing the mean values of the estimated log probabilities for each K-s, a consistent pattern was present until the K = 6 (Additional file 8), while a significant drop was detected at K = 8. Following the recommendations [32, 34, 40] based on the ΔK, the most likely number of K was 2 (Additional file 8), which is highly expected as two subspecies were clearly separated into two genetic clusters. The next most likely model was K = 3 and in this scenario, we observed clear separation of AML from the AMC populations, along with the assignment of AMC into two clusters. At assumed K = 3, two populations which had > 90% of the genetic ancestry assigned to the cluster 2, were SLO and CROS, while on the other hand, the MNE and CROA populations had 60% and 32%, of their genetic ancestry assigned to cluster 3, respectively. Despite their relatively large geographical distribution in Central Europe, admixture is present between all AMC populations. Genetic admixture was detected in K = 4 and K = 5 to a lesser degree between the Croatian, Slovenian, and Hungarian populations, while the highest admixture was obtained in CROA and SRB, BIH and especially in MNE populations. Given that Croatia encompasses a wide range of distinct environments - including continental, alpine, and coastal Adriatic regions - each plays a crucial role in the adaptation of honey bees. We collected 103 samples exclusively from Croatia to capture this environmental diversity. The DAPC results indicate that the two subpopulations or “ecotypes” (CROC and CROS) previously thought to exist within Croatia [41, 42] are in fact a single genetically unified population. The Adriatic population (CROA), while slightly more dispersed and admixed with third cluster at K = 3, does not exhibit considerable genetic differentiation from the other Croatian population (CROC and CROS). This finding suggests that the genetic distinctions within these groups are minimal, reflecting a cohesive genetic structure across diverse ecological zones. Such insights can have implications for future conservation and breeding programs, as they highlight the need to manage these populations as a unified genetic entity while considering their slight regional adaptations. This unified approach can ensure that the bees remain well-adapted to the varying environmental conditions throughout the Croatian territory.

#### Gene flow

**Fig. 5:**
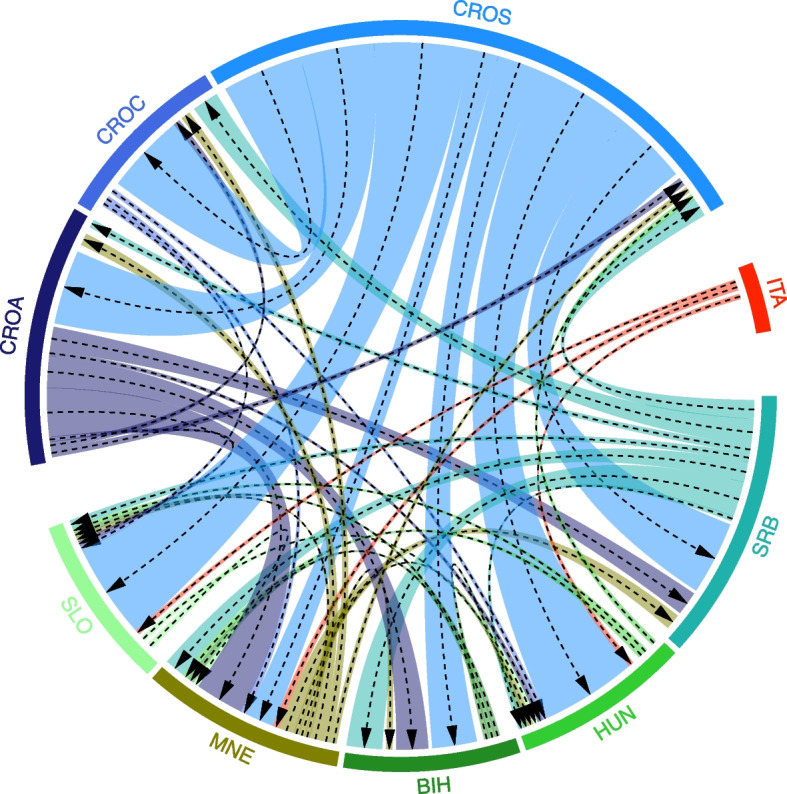
Estimated relative migration pattern between one AMC population (ITA - Italy) and eight AML populations (CROA - Adriatic Croatia; CROC - continental Croatia; CROS - subalpine Croatia; BIH - Bosnia and Herzegovina; HUN - Hungary; MNE - Montenegro; SLO - Slovenia and SRB -Serbia). The strongest migrations (> 1%) are marked with the gene flow directions by the black dashed arrows. The thicker area around the lines indicates the direction of gene flow for > 1, > 3, > 5 and > 10%

The contemporary migration rate (gene flow) shows that the recent migration rate between the populations studied was characteristic of all populations to some degree (Fig. 5), regardless of whether it was “in-migration” or “out-migration”. Most out-migration was found from the CROS population to most other AMC populations. Populations that also showed a high migration rate to other AMC populations were CROA and SRB. Unexpectedly, no considerable gene flow was observed from the SLO population, instead only a gene flow of slightly more than 1% towards MNE and HUN was detected.

The surprisingly low out-migration to the CROA area could be due to certain natural barriers, such as mountains, restricting in-migration to the area, while the “out-migration” from the CROA area is most likely due to anthropogenic factors favoured by the early development of the colonies. The populations from MNE and BIH showed the highest gene flow from various other populations. AML from ITA showed low gene flow (> 1%) to other populations (SLO, MNE and HUN), which in the case of SLO could be due to direct migration from Italy, whereas the out-migration to MNE and HUN is more likely due to anthropogenic factors. At the same time, no migration from AMC to AML was observed. The results of the migration analyses (gene flow) are consistent with the results of the admixture.

#### Isolation-by-distance

**Fig. 6:**
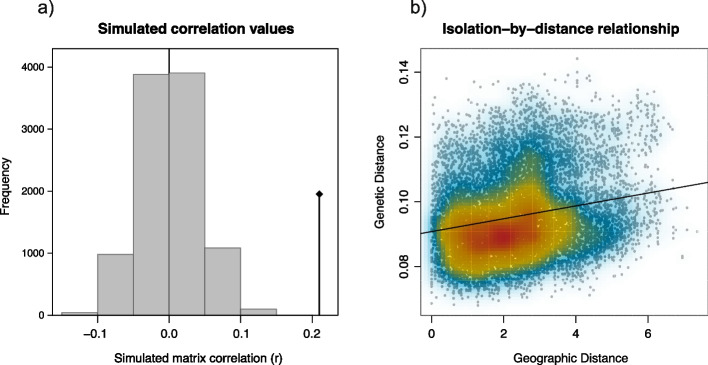
Results of Isolation-by-distance analysis by using Mantel test. **a** Histogram of simulated correlation values using permutation. Correlation between the Nei’s genetic distance and geographic distance matrices is represented by the vertical line and black dot. **b** Scatterplot of the density of population samples plotted using a two-dimensional kernel density estimate. The linear regression trend is shown by black line while colours represent degrees of density (blue: low; yellow: medium; red: high density)

We conducted an Isolation-by-distance analysis using the Mantel test (Fig. 6a and b). The correlation between the genetic and geographic matrices was r = 0.21, indicating a slight linear relationship between genetic and geographic distances. The two-dimensional kernel density plot (Fig. 6b) illustrates this relationship with a single, consistent cloud of data points. A permutation test (*p* = 0.001; assuming the absence of spatial structure) resulted in the original correlation coefficient value being outside the simulated distribution (Fig. 6a), thus indicating a significant effect of Isolation-by-distance.

## Discussion

The honey bee subspecies AMC is the second most common honeybee subspecies after AML [43]. It has spread through trade and human migration over all continents except Antarctic. The exchange of honey bee genetic material is contributing to the decline of honeybee populations that have evolved to thrive in their local environment, which is a significant concern for the preservation of honeybee diversity [44]. Our research investigated the current status of the genetic pool of AMC population in part of Central and South-Eastern European countries, covering a continuous area of the native Carniolan honey bee habitat.

Conservation of the local gene pool was found as paramount: it was shown that colonies of the local genotype respond better to challenges in comparison with imported ones [41, 45–48]. Traditionally, morphometry was employed to address the biodiversity of *A. mellifera* in Europe [12, 49], often finding statistically significant differences between populations in selected traits. On the other hand, values of certain traits can overlap in some populations offering no clear resolution between subspecies (c. f. cubital index between AMC and AML [12]).

We have analysed extensive data of honey bees from seven countries representing continuous areal using comprehensive set of genetic analyses, including Discriminant analysis of principal components (DAPC), analyses of heterozygosity and population inbreeding (F), admixture analysis, analysis of F_ST,_ Nei’s genetic distances, gene flow and Isolation-by-distance to elucidate the genetic structure, differentiation, and relationships among honey bee populations based on single nucleotide polymorphism (SNP) data.

DAPC is an exploratory analysis that can reveal hidden patterns in the genetic data. The results of obtained SNPs revealed clear separation between the AML and AMC populations in the studied areal, which is consistent with their distinct subspecies status (AML vs AMC). The first and second discriminant functions accounted for a significant proportion of the total variation, highlighting the differences between these two groups. The separation was also supported by pairwise F_ST_ values and Nei’s genetic distances. According to traditional perceptions, the Slovenian population is believed to be situated at the edge of the AMC geographic distribution [12]. However, the further dimensions of discriminant functions showed homogeneity among AMC populations, including the Slovenian population which seemed to be the most homogenous. Also, these findings are consistent with large morphometric analysis [14] on the same dataset. To further explore the genetic structure and admixture patterns among the populations, an admixture analysis was conducted. The first split observed at K = 2, separating the AMC and AML populations, was expected due to their different subspecies status. Despite their close geographical proximity, only a minimal level of admixture was detected, which was also confirmed with low gene flow (> 1%) between these two groups. Thus, the higher-level conservation status of AMC at the studied territory seem to be intact. Within the AMC populations, admixture was found to a lesser extent between the Croatian, Slovenian and Hungarian populations. As some studies indicate the existence of AMC ecotypes within Croatia [41, 42] based on our results, the AMC population should be considered as a specific and valuable single population with a certain degree of genetic variation between CROA, CROC and CROS. On the other hand, the Adriatic population has shown a higher level of genetic admixture and specific directional gene flow to populations from Bosnia and Herzegovina, Montenegro and Serbia, which is probably due to anthropogenic factors. We were surprised that CROA and MNE were found to be slightly divergent, although DAPC analysis revealed some neighbouring similarities between these two populations (Fig. 2b). The native areal of AMC is very heterogenous in terms of environment and climate. Based on Köppen—Geiger climate classification it ranges from Cf* (temperate, no dry season), Cs* (temperate, dry summer) to Df* (Continental, no dry season) with “*” symbol denoting temperatures [50]. The Adriatic coast, for example, is comparable to the environment in Apennine peninsula, but it switches to C type of climate with landscape rapidly gaining altitude when going away from the sea. In the evolutionary perspective, one speculates that adaptations in coastal region should differ from those in continental background. The speculative reason could be colony migrations when beekeepers are trying to catch nectar flows at different points of their countries. Other reason could be normal commerce, purchasing queens and swarms outside of local population. Any kind of genetic migrations—either through movement of colonies or through purchase of queens or swarms—between coastal regions and inland would result in admixture pattern such as detected. Additional support for this is the direction of gene flow, which goes from Croatian Adriatic population to Montenegro, Bosnia and Herzegovina and Serbian population. Diversity of population in Adriatic region was also confirmed [16]. They identified two subpopulations in the Adriatic region; however, due to the limitations of the markers used, smaller sample sizes, and a narrower scope of populations analysed, they were unable to interpret the results in a broader context.

The analysis of heterozygosity and population inbreeding coefficients provided further insights into the genetic diversity within the honey bee populations. The Montenegro population exhibited the highest level of genetic diversity, as evidenced by the highest observed heterozygosity and lowest F_POP_ value and this suggests a greater genetic variability. In contrast, the Italian population displayed the lowest diversity, indicating a lower variability. The Slovenian, Croatian, Hungarian, and Serbian populations exhibited similar levels of genetic diversity, characterized by relatively moderate values of observed heterozygosity and F_POP_. The negative F_POP_ values observed in populations such as Serbia, Hungary, and Bosnia and Herzegovina may imply an excess of heterozygosity compared to the expected values, indicating genetic diversity within these populations resulting from gene flow, which was detected in this study, or outbreeding [28]. However, given the uncertainty related to these estimates, it is crucial for ensuring robust conclusions from our findings. We hypothesize that larger and more representative sample sizes (especially from northern Hungary and eastern Serbia) would offer greater confidence in calculating population inbreeding coefficients.

To assess population divergence and relationships, pairwise F_ST_ values were calculated. The mean F_ST_ estimate across all honey bee populations, including the AML, indicated a moderate level of genetic differentiation. However, when considering only the AMC populations, the mean F_ST_ value was lower, indicating a closer genetic relationship among these populations. The pairwise F_ST_ values ranged from zero (between the Serbian and Bosnian populations) to 0.359 (between the Montenegro population and AML). Generally, genetic differentiation tends to be lower between populations with closer genetic history, which aligns with the quite low F_ST_ values observed among the AMC populations in this study.

Neighbour-Joining tree provided further insights into the level of genetic divergence between investigated honey bee populations based on the allele frequencies of analysed SNP markers. When allele frequencies are similar in two populations, Nei’s identity *I* (or simply genetic similarity) approaches 1, while the genetic distance *D* approaches zero, and vice versa. This measure of distance *D* reflects accumulated allele differences per each SNP locus as a result of mutation and drift, and it is also linearly related to the divergence time [51]. Our results showed quite low values among AMC populations, ranging from 0.002 (between CROS and CROC) to 0.020 (between SLO and MNE), respectively. In our scenario on AMC, if we assume stable rate of genetic change over time for all examined loci, low genetic distances could indicate that AMC populations share a relatively recent common ancestry. Contrary, if they diverged more anciently, they would have accumulated many genetic differences. Other arguments which support this scenario are continuous gene flow and stable environment. There was a constant gene flow between the populations due to the colony migrations (supported by our gene flow results, admixture results and Isolation-by-distance), preventing significant genetic differentiation. Also, if the environment for honey bee populations was relatively stable, there might not have been strong selective pressures leading to significant genetic changes [52]. Our gene flow results confirm this idea of gene flow going in all directions as its magnitude is in concordance with detected genetic admixture. The only surprise for us was the CROS, from which the highest gene flow (in both magnitude and dispersion) was detected compared to all other populations. One possible explanation could be that it is located in the central region of the AMC geographical distribution of this sample, from which migration occurs. It is also important to note that calculated gene flow reflects recent migrations, which, as previously mentioned, in the context of honey bees, is influenced by both natural migrations and human activities. In our dataset, populations are categorised a priori according to countries, which is logical only in the context of human influence and less in relation to the geographical characteristics of the area (as previously discussed, the AMC area is quite heterogeneous). Therefore, we also conducted an Isolation-by-distance analysis. Plotting genetic distance among populations against their geographic distances revealed a slight yet significant correlation. This suggests that more distant populations are also more genetically distant, albeit to a very small degree (r = 0.21), as determined by the Mantel test. When the Mantel correlation coefficient approaches 1, an increase in geographic distance between populations strongly corresponds to an increase in genetic distance. Hence, based on our findings, we can infer that the genetic divergence of AMC populations is influenced by spatial distance, albeit to a minimal extent.

The AML population as expected, displayed higher genetic distances from all AMC populations (average 0.043), indicating a greater level and more ancient genetic divergence between the AML and AMC. Contrary to our results, genetic analyses based on microsatellites usually reports higher Nei’s distances among populations within the same honey bee species [53, 54], due to their higher mutation rate and usually low number of markers used in studies [55].

Diversity within a population is beneficial for breeding programs, as it provides a broad genetic pool for sourcing specific desirable traits, such as disease resistance or overall genetic diversity needed for a robust selection response. Based on our study, we recommend the following for implementing effective breeding programs; i) Maintain Current Diversity: Regularly monitor the population’s genetic diversity. Studies like this one offer a snapshot of the current genetic landscape. Since populations are dynamic, frequent snapshots are necessary to ensure proper management and conservation. Honey bee breeding organizations should facilitate the genotyping of breeding colonies to prevent high inbreeding and maintain diversity. ii) Enhance Selection Methods: Improve current selection methods based on phenotype and pedigree records to lay the groundwork for future genomic evaluations. This approach is expected to increase the accuracy and reliability of estimated breeding values, enhance genetic gain, and provide a solid foundation for maintaining genetic diversity.

In a recently published study [56], a solution for the implementation of the genomic selection scheme of the AMC honey bee in Germany based on a genome-wide 100 k SNP chip was presented. Considering the fact that after the introduction of genomic selection more than ten years ago in various livestock species, on the one hand populations were significantly improved in terms of production traits [57], on the other hand genetic diversity was dramatically reduced, especially when parameters such as inbreeding and effective population size are taken into account [58, 59]. These effects should also be considered in future honey bee breeding programs. Therefore, further research should focus on exploring additional genetic markers, evaluating the functional significance of genetic differentiation, e.g. of key traits such as honey yield or disease resistance, and investigating the influence of environmental factors on population dynamics. Such findings could provide additional valuable insights for the sustainable management and conservation of honey bee populations to ensure their resilience and contribution to ecosystem health and pollination services.

## Conclusions

The genetic analyses of AML and several AMC populations from Central and South-Eastern Europe conducted in this study provided valuable insights into the genetic structure, differentiation and relationships between honey bee populations based on genome-wide SNP data. DAPC analysis revealed a clear separation between the AMC and AML populations, while the admixture and migration analyses demonstrated varying degrees of gene flow and genetic admixture between AMC populations, with closer relationships observed between specific geographic locations, as confirmed by isolation by distance analysis. Analysis of heterozygosity, population inbreeding coefficients, pairwise F_ST_ values and Neighbour-Joining tree analysis further clarified patterns of genetic differentiation and similarity between populations. Thus, we found that the AMC population functions as a metapopulation in a broader genetic context. Within its original geographic range, the AMC metapopulation exhibits sufficient genetic diversity, with notable genetic clines and the greatest differentiation observed between the Slovenian and Montenegrin populations. This genetic diversity needs careful monitoring and maintenance. Overall, these results enhance our understanding of the genetic diversity, population dynamics, and evolutionary history of honey bees, which has significant implications for conservation measures, breeding programs, and sustainable beekeeping practices.

## Supplementary Information

Supplementary Material 1.

Supplementary Material 2.

Supplementary Material 3.

Supplementary Material 4.

Supplementary Material 5.

Supplementary Material 6.

Supplementary Material 7.

Supplementary Material 8.

## Data Availability

SNP data used in this study have been deposited in the European Nucleotide Archive with the primary accession code PRJEB79457.

## Abbreviations

DAPC: Discriminant analysis of principal components
AMC: *Apis mellifera carnica*
AML: *Apis mellifera ligustica*
CROC: Croatian continental region
CROS: Croatian subalpine region
CROA: Croatian Adriatic region
SLO: Slovenia
SRB: Serbia
BIH: Bosnia and Herzegovina
MNE: Montenegro
HUN: Hungary
ITA: Italy
