# Supplementary figures and images for: Genomic diversity and population structure of Carniolan honey bee in its native habitat
Source: BMC Genomics. 2024 Sep 10;25:849. doi: 10.1186/s12864-024-10750-z (PMC11385169; doi:10.1186/s12864-024-10750-z)

# Honeybee 4K SNPchip – The number of SNPs within 1Mb window size

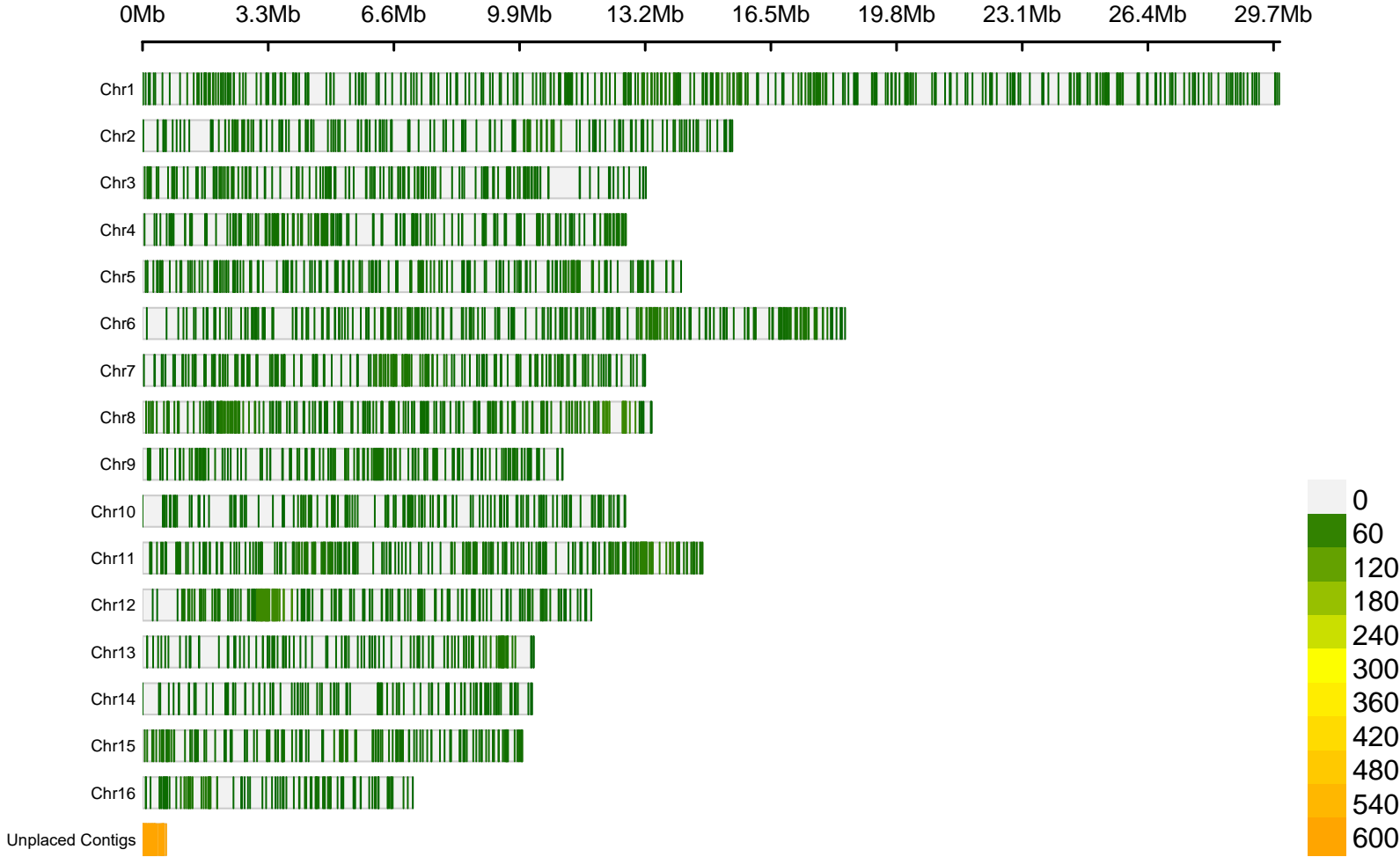

Supplement: Supplementary file 2 — Supplementary Material 2. [file 12864_2024_10750_MOESM2_ESM.pdf]

10 retained clusters

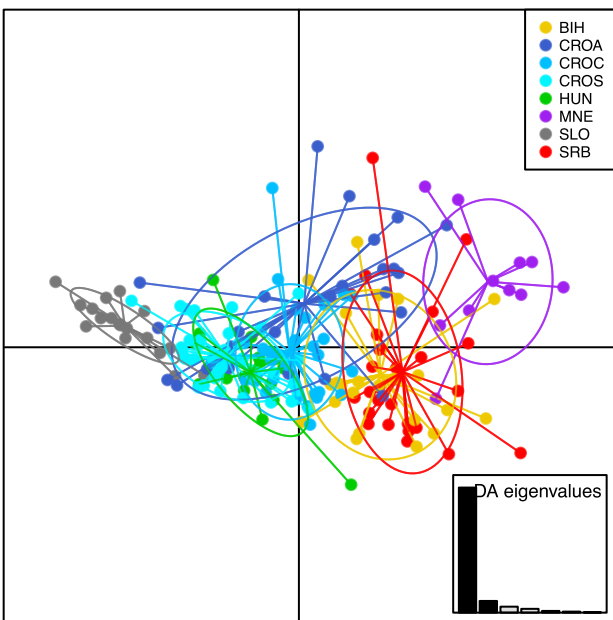

30 retained clusters

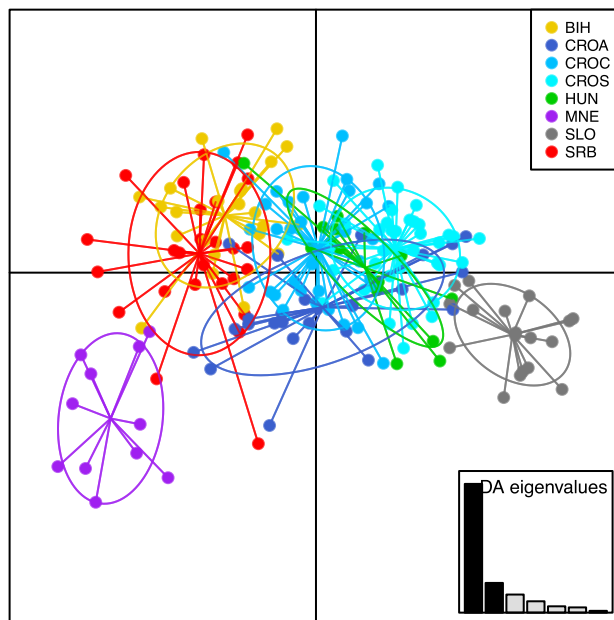

50 retained clusters

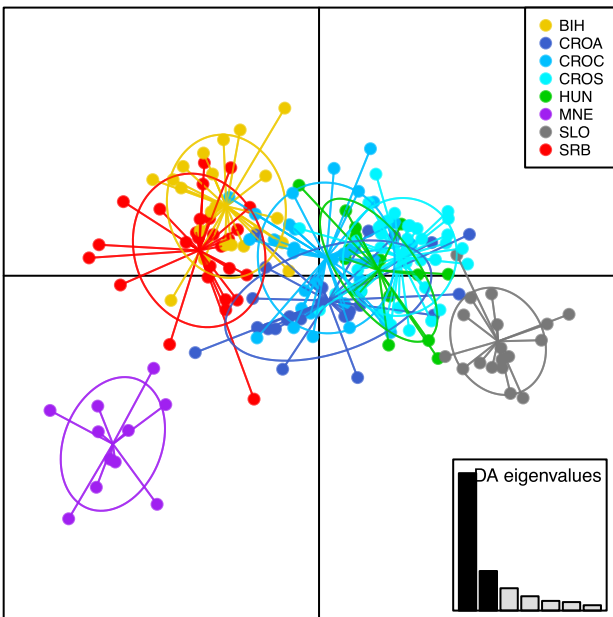

100 retained clusters

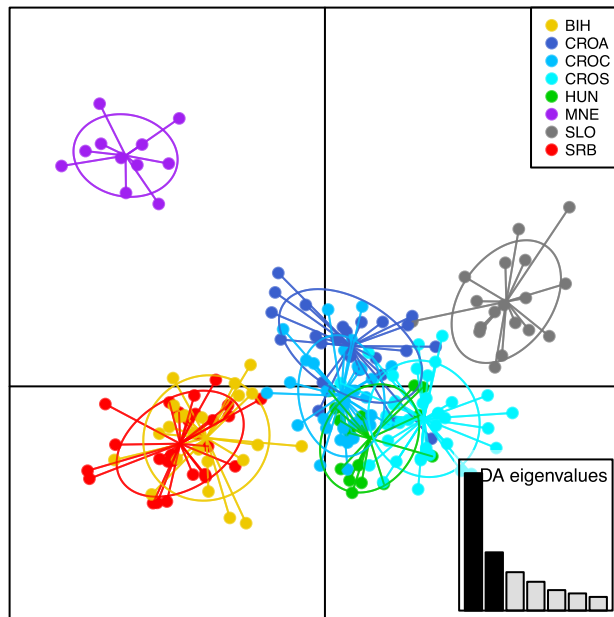

Supplement: Supplementary file 4 — Supplementary Material 4. [file 12864_2024_10750_MOESM4_ESM.pdf]

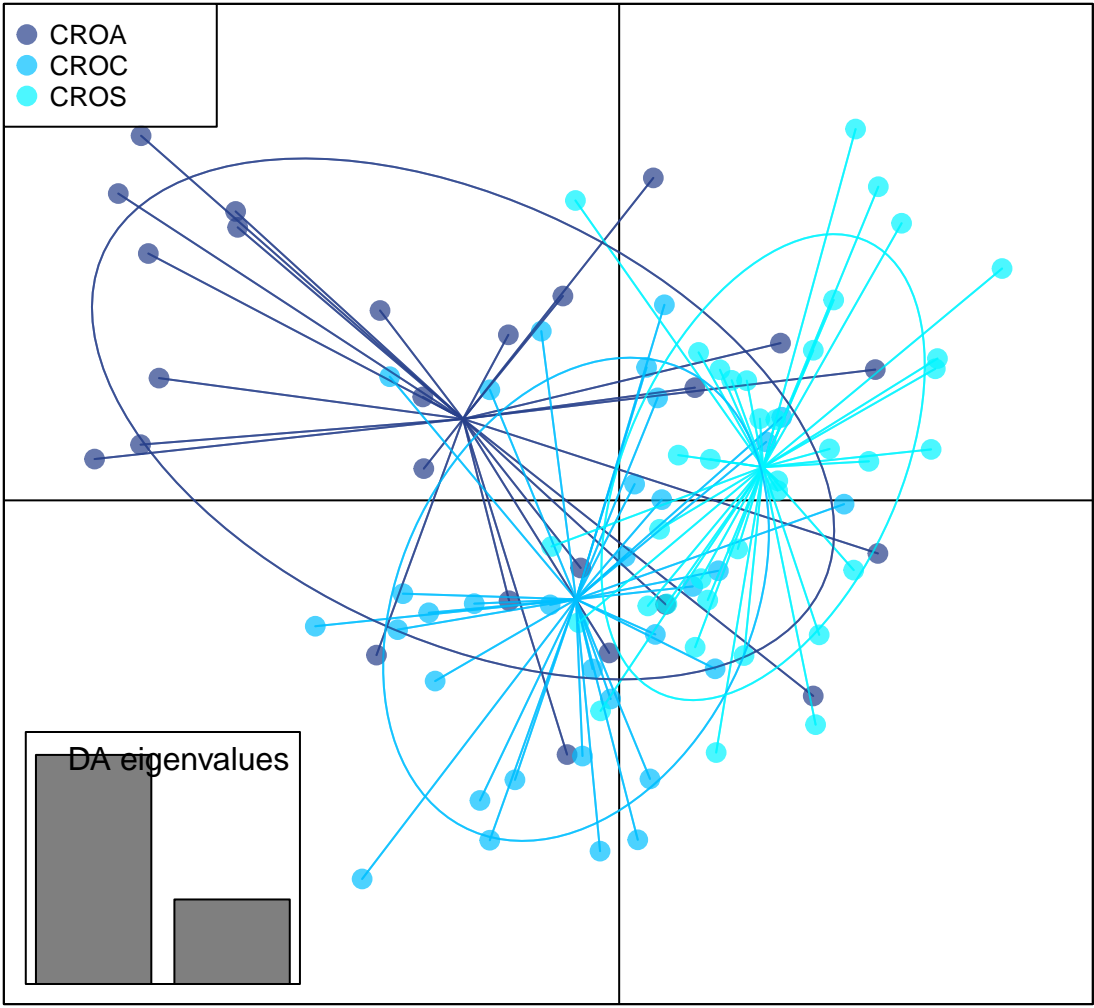

Supplement: Supplementary file 7 — Supplementary Material 7. [file 12864_2024_10750_MOESM7_ESM.pdf]

a)

Mean LnP (K)  $\pm$  Stdev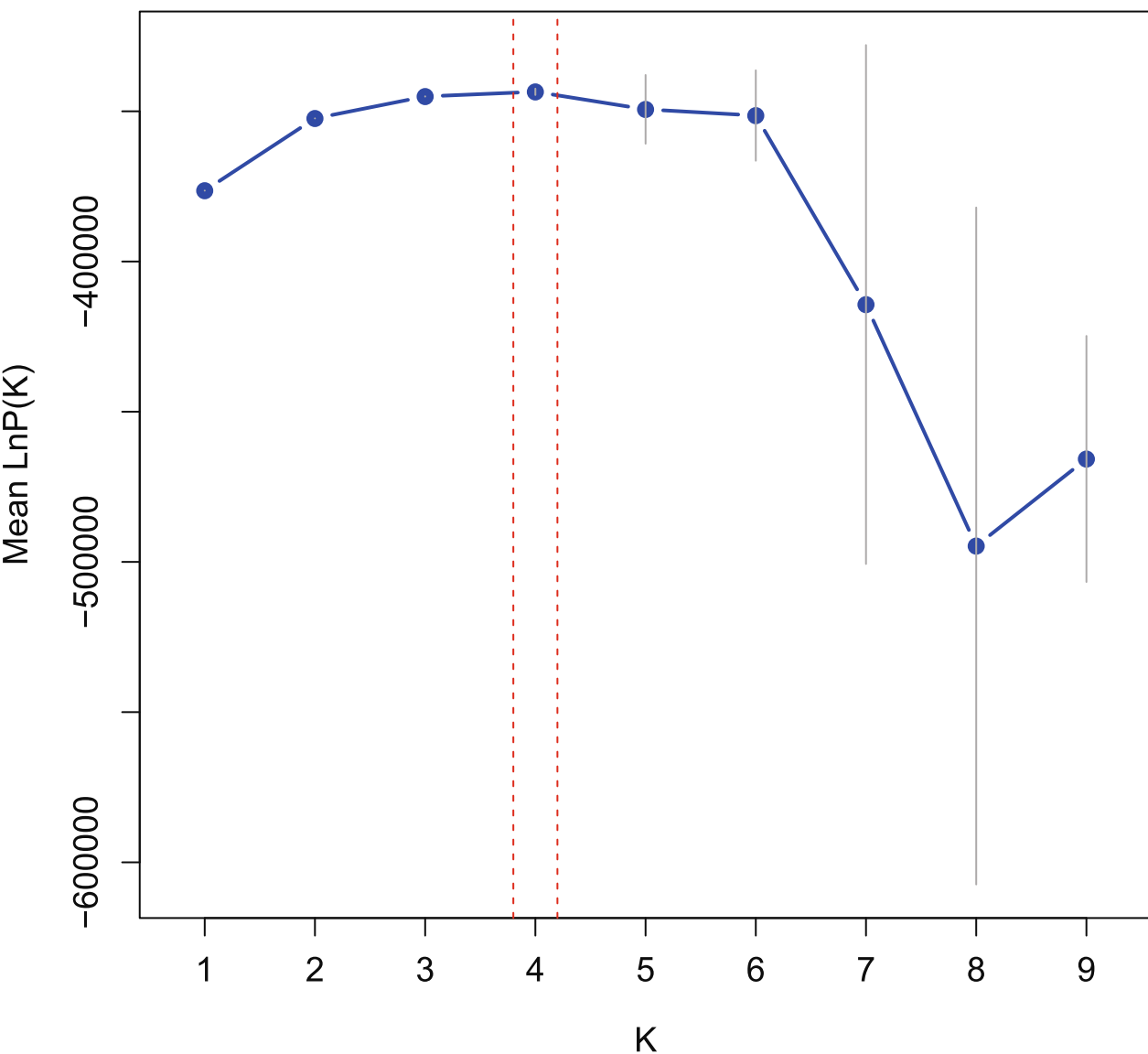

b)

Delta K

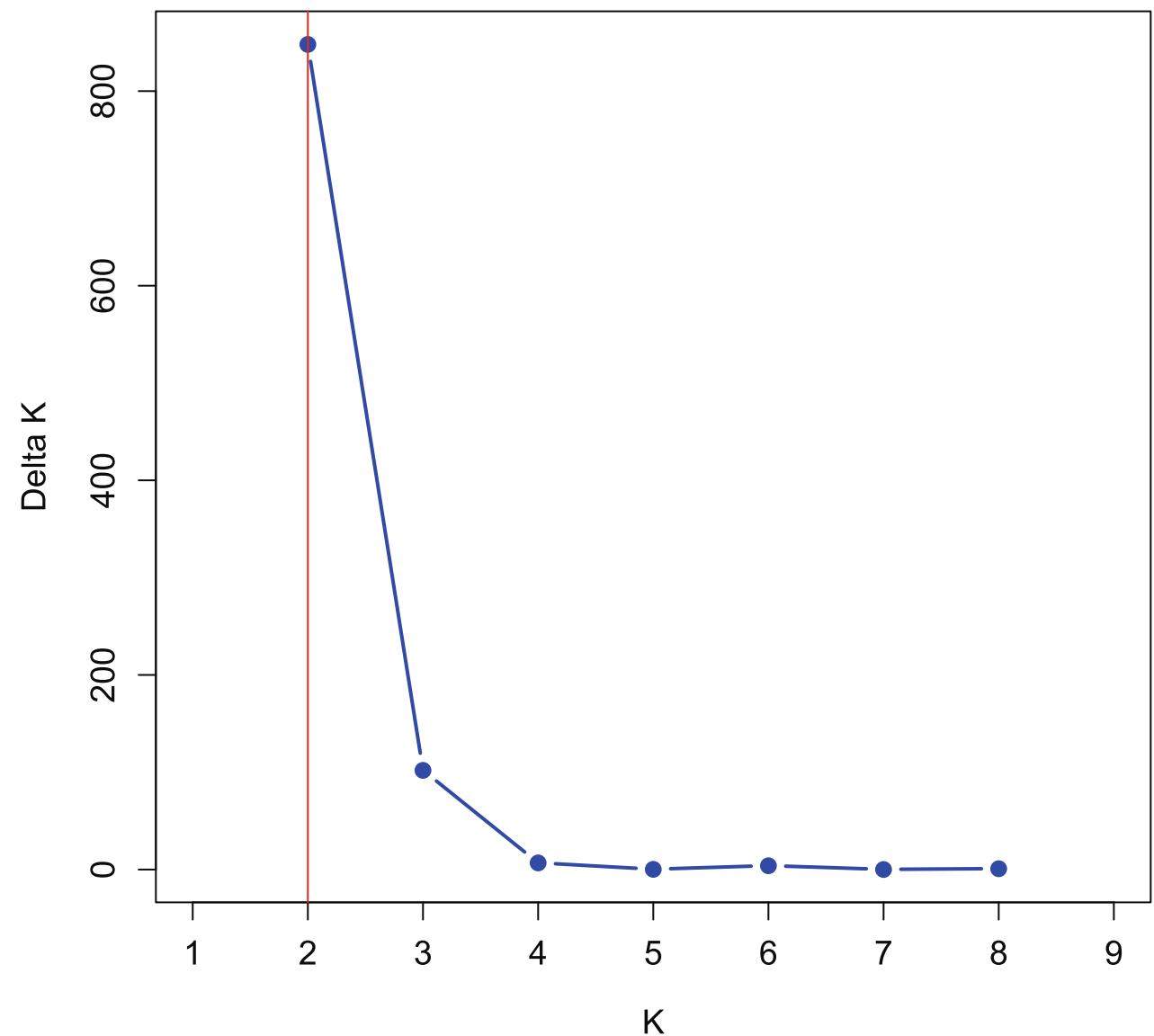

Supplement: Supplementary file 8 — Supplementary Material 8. [file 12864_2024_10750_MOESM8_ESM.pdf]
