# Supplementary material for: Genomic diversity and population structure of Carniolan honey bee in its native habitat
Source: BMC Genomics. 2024 Sep 10;25:849. doi: 10.1186/s12864-024-10750-z (PMC11385169; doi:10.1186/s12864-024-10750-z)
Supplement: Supplementary file 3 — Supplementary Material 3. [file 12864_2024_10750_MOESM3_ESM.pdf]

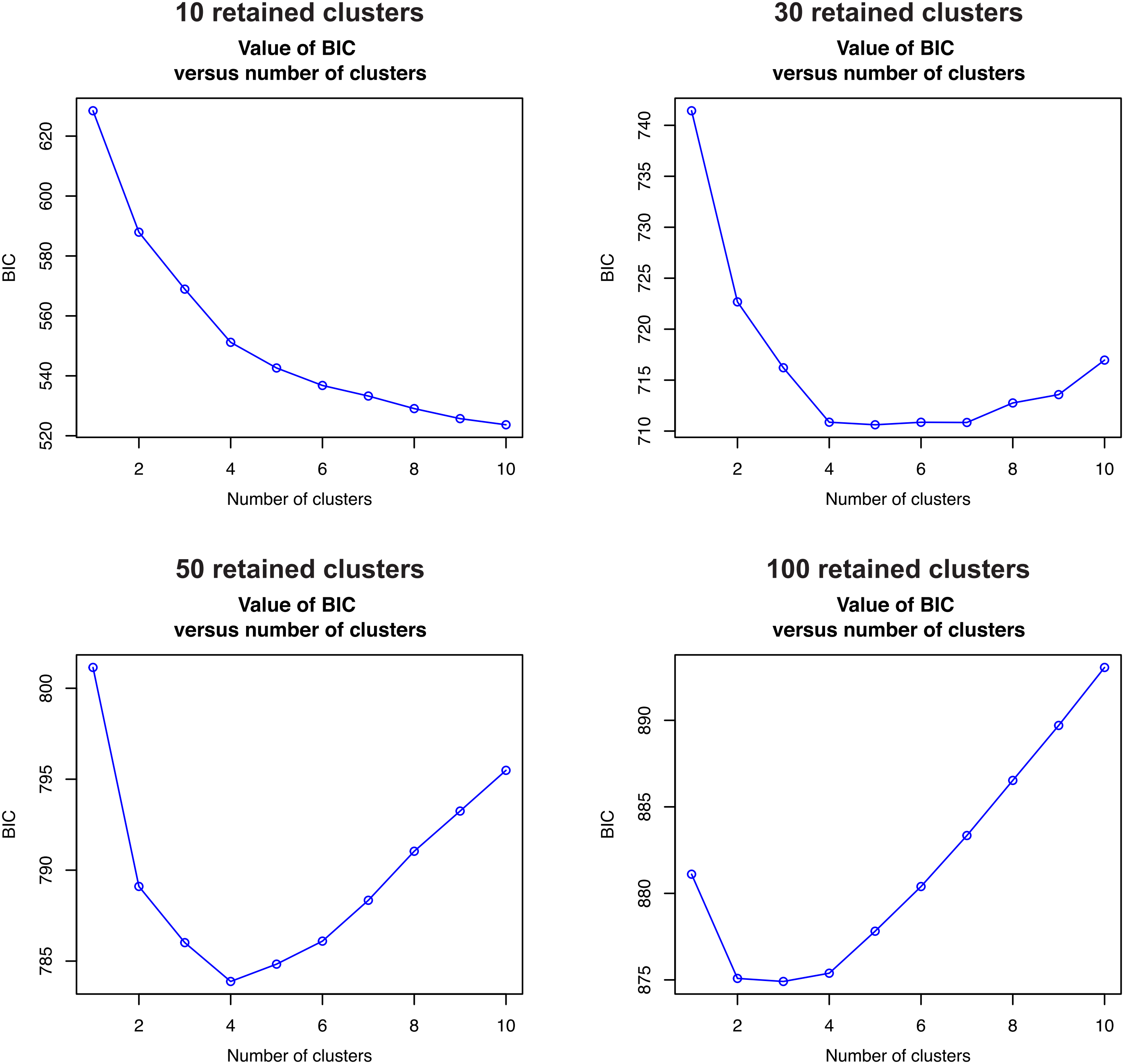
10 retained clusters
Value of BIC
versus number of clusters
BIC
520
540
560
580
600
620
2
4
6
8
10
Number of clusters
30 retained clusters
Value of BIC
versus number of clusters
BIC
710
715
720
725
730
735
740
2
4
6
8
10
Number of clusters
50 retained clusters
Value of BIC
versus number of clusters
BIC
785
790
795
800
2
4
6
8
10
Number of clusters
100 retained clusters
Value of BIC
versus number of clusters
BIC
875
880
885
890
2
4
6
8
10
Number of clusters
