# Supplementary material for: Genomic diversity and population structure of Carniolan honey bee in its native habitat
Source: BMC Genomics. 2024 Sep 10;25:849. doi: 10.1186/s12864-024-10750-z (PMC11385169; doi:10.1186/s12864-024-10750-z)
Supplement: Supplementary file 5 — Supplementary Material 5. [file 12864_2024_10750_MOESM5_ESM.pdf]

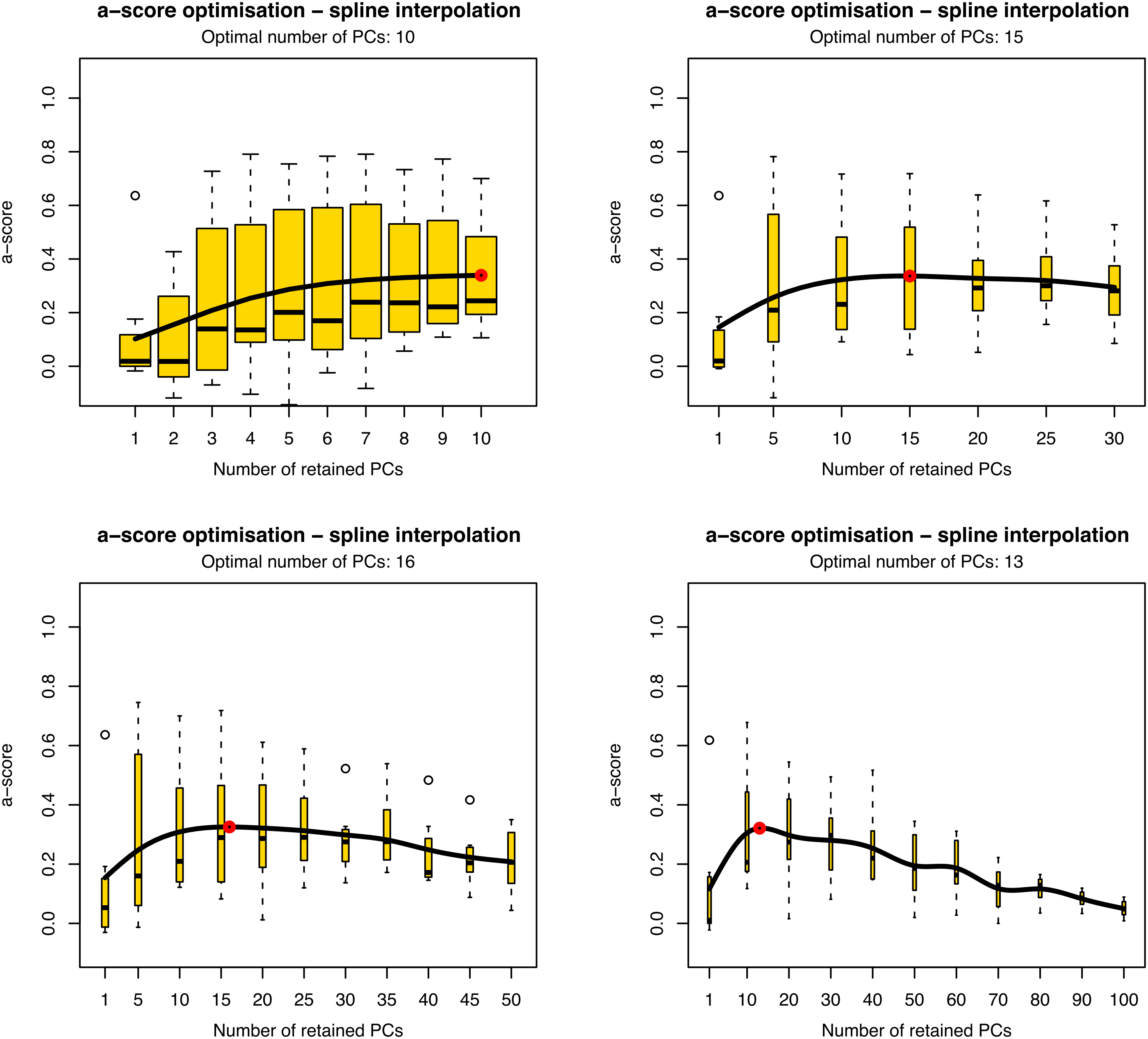
a–score optimisation – spline interpolation
Optimal number of PCs: 10
a–score
Number of retained PCs
a–score optimisation – spline interpolation
Optimal number of PCs: 15
a–score
Number of retained PCs
a–score optimisation – spline interpolation
Optimal number of PCs: 16
a–score
Number of retained PCs
a–score optimisation – spline interpolation
Optimal number of PCs: 13
a–score
Number of retained PCs
